# Electrophysiological Characterization of Human Atria: The Understated Role of Temperature

**DOI:** 10.3389/fphys.2021.639149

**Published:** 2021-07-23

**Authors:** Rupamanjari Majumder, Afnan Nabizath Mohamed Nazer, Alexander V. Panfilov, Eberhard Bodenschatz, Yong Wang

**Affiliations:** ^1^Laboratory for Fluid Physics, Pattern Formation and Biocomplexity, Max Planck Institute for Dynamics and Self-Organization, Göttingen, Germany; ^2^DZHK (German Center for Cardiovascular Research), Partner Site Göttingen, Göttingen, Germany; ^3^Cardiovascular Science, University Medical Center Göttingen, Göttingen, Germany; ^4^World-Class Research Center “Digital Biodesign and Personalized Healthcare”, Sechenov University, Moscow, Russia; ^5^Department of Physics and Astronomy, Ghent University, Ghent, Belgium; ^6^Laboratory of Computational Biology and Medicine, Ural Federal University, Yekaterinburg, Russia; ^7^Laboratory of Atomic and Solid-State Physics and Sibley School of Mechanical and Aerospace Engineering, Cornell University, Ithaca, NY, United States

**Keywords:** cardiac electrophysiology, temperature, remodeling, human atria, atrial fibrillation, action potential, restitution

## Abstract

Ambient temperature has a profound influence on cellular electrophysiology through direct control over the gating mechanisms of different ion channels. In the heart, low temperature is known to favor prolongation of the action potential. However, not much is known about the influence of temperature on other important characterization parameters such as the resting membrane potential (RMP), excitability, morphology and characteristics of the action potential (AP), restitution properties, conduction velocity (CV) of signal propagation, etc. Here we present the first, detailed, systematic *in silico* study of the electrophysiological characterization of cardiomyocytes from different regions of the normal human atria, based on the effects of ambient temperature (5−50°*C*). We observe that RMP decreases with increasing temperature. At ~ 48°*C*, the cells lose their excitability. Our studies show that different parts of the atria react differently to the same changes in temperature. In tissue simulations a drop in temperature correlated positively with a decrease in CV, but the decrease was region-dependent, as expected. In this article we show how this heterogeneous response can provide an explanation for the development of a proarrhythmic substrate during mild hypothermia. We use the above concept to propose a treatment strategy for atrial fibrillation that involves severe hypothermia in specific regions of the heart for a duration of only ~ 200 ms.

## 1. Introduction

Abnormal generation and propagation of electrical signals in the heart lead to disturbances in its regular electromechanical pump function. Such disturbances can occur abruptly over a few beats and disappear by themselves, or they can persist for a sufficiently long period of time to be classified as an arrhythmia. Cardiac arrhythmias can be of different types. The most lethal variants include ventricular fibrillation (VF), ventricular tachycardia (VT), and atrial fibrillation (AF), of which persistent AF has the highest rate of occurrence in human patients (Nattel, 2002; Nattel et al., 2008; Andrade et al., 2014). Unlike VF, VT, and AF are treatable by pharmacological methods (Ravens et al., 2013) or by other means, such as radiofrequency (RF) ablation (Hussein et al., 2016). The method involves destruction of excitable heart tissue to *(i)* remove the “source” of the arrhythmia, and *(ii)* to prevent recurrence at the same site. Despite its high success rate, RF ablation involves a major compromise, namely the infliction of permanent damage to healthy heart tissue. Therefore, alternative, low-energy defibrillation methods, are in great demand. In particular, recently, optogenetics has emerged as a promising tool to study and control wave dynamics in cardiac tissue (Bruegmann et al., 2010; Entcheva, 2013; Bingen et al., 2014; Nussinovitch and Gepstein, 2015; Nyns et al., 2019; O'Shea et al., 2019). However, despite its success in the treatment of arrhythmias in small mammalian hearts (Bruegmann et al., 2010; Bingen et al., 2014; Majumder et al., 2018; Nyns et al., 2019), optogenetics suffers from two major limitations: (*i*) the long term effects of genetic modification are not completely known, which makes clinical translation debatable; and (*ii*) penetration of light in cardiac tissue is very poor, making it difficult to defibrillate deep tissue (Bruegmann et al., 2016).

Ambient temperature (*T*) has long been known to influence cellular and molecular processes (Hodgkin and Katz, 1949; Hannon, 1958; Bjornstad et al., 1993; Kiyosue et al., 1993; Zhao and Boulant, 2005; Kagstrom et al., 2012). In particular, temperature has a direct bearing on ion channel kinetics, through its influence on the rate constants of gating, and reaction rates of certain sub-cellular chemical processes, which thereby allow cells to produce action potentials with altered morphology, characteristics and cell-to-cell propagation speeds. In cardiac electrophysiology, the effect of temperature is modeled by dividing the time constant for opening/closing of an ion channel, by a temperature-dependent scaling factor *Q*(*T*), [or equivalently, multiplying the rate constants of the relevant sub-cellular chemical reaction, by *Q*(*T*)]. *Q*(*T*) has a power-law dependence on temperature, according to the Arrhenius law of heat transfer (Hodgkin and Katz, 1949; Malki and Zlochiver, 2018). Previous studies showed that in small mammals such as the rabbit and ground squirrel, lowering temperature leads to depolarization of the resting membrane potential (RMP), reduction in action potential amplitude (APA), and prolongation of action potential duration (APD) at 50, 70, and 90% repolarization, at the single cell level (Fedorov et al., 2005), whereas, in tissue, signal conduction velocity (CV) was found to decrease with decrease in temperature (Fedorov et al., 2008). These electrophysiological behavior are similar to those obtained with cardiac optogenetics. However, unlike optogenetics, here the cells do not require genetic modification.

In a recent study, Tobón et al. (2013) presented a model for anatomically realistic human atria, based on geometry and fiber data acquired using Diffusion Tensor Magnetic Resonance Imaging (DTMRI). Their model characterizes the atria while taking into account, the differences in action potential morphology and characteristics, and conduction anisotropy, as measured from different regions of the atria.

Here, we developed a model of the electrophysiologically-detailed human atria, based on the Courtemanche-Ramirez-Nattel (CRN) model for single cell action potentials (AP) from human atrial cardiomyocytes (Courtemanche et al., 1998), using the AP-characteristic data that was used to develop the Tobón model (Tobón et al., 2013). We subdivided the atria into the following regions: Crista terminals (CT), Pectinate Muscles (PM), Right and Left atrial appendages (APG), Right and Left atrioventricular regions (AVR) and the remaining atrial working myocardium (AWM) and incorporated temperature sensitivity to its different parts. In particular, we incorporated temperature dependence into the kinetics of the different ion channels in CT, PM, APG, AVR and AWM regions. We used it to demonstrate low-temperature induced break-up of scroll waves during mild hypothermia consistent with the results of Filippi et al. (2014) for canine hearts, and the low-temperature induced suppression of scroll waves during severe hypothermia. Our finding leads us to consider thermal control as a possible tool for arrhythmia management in the future.

## 2. Materials and Methods

We modeled the time evolution of the transmembrane potential *V* according to Equation (1), where we described the net ionic current (*I*_*ion*_) flowing across the cell membrane, according to the CRN model (Courtemanche et al., 1998) for human atrial cardiomyocytes. There are several different models available for human atrial cardiomyocytes. We picked the CRN model because its accuracy has been found to be quite standard with respect to other models, while dealing with fewer parameters. By standard, we mean in terms of its ability to reproduce the APD restitution curve, the general AP charateristics: its upstroke, duration, amplitude, resting membrane potential, excitation threshold etc. and the model's ability to allow the atria to be stimulated at very high frequencies such as those observed during AF. For a comparison of different mathematical models used to study the human atria, we refer the readers to Wilhelms et al. (2013).

(1) $$\begin{array}{l} \frac{dV}{dt}=-\frac{I_{ion}+I_{stim}}{C_{m}} \end{array}$$

Here, *C*_*m*_ is the specific capacitance of the cell membrane (in μF/cm^2^). As given in Equation (2), *I*_*ion*_ represents the sum of 12 ionic currents: the fast *Na*^+^ current (*I*_*Na*_), the inward rectifier *K*^+^ current (*I*_*K*1_), the transient outward *K*^+^ current (*I*_*to*_), the ultra-rapid *K*^+^ current (*I*_*Kur*_), the rapid and slow delayed rectifier *K*^+^ currents (*I*_*Kr*_ and *I*_*Ks*_, respectively), the *Na*^+^ and *Ca*^2+^ background currents (*I*_*BNa*_, and *I*_*CaL*_), the *Na*^+^/*K*^+^ pump current, the *Ca*^2+^ pump current (*I*_*pCa*_), the *Na*^+^/*Ca*^2+^ exchanger current (*I*_*NaCa*_) and the *L* − *typeCa*^2+^ current (*I*_*CaL*_).

(2) $$\begin{array}{l} I_{ion}=I_{Na}+I_{K1}+I_{to}+I_{Kur}+I_{Kr}+I_{Ks}+I_{BNa}+I_{BCa}+I_{NaK}\\ \;+I_{CaP}+I_{NaCa}+I_{CaL} \end{array}$$

We used forward Euler method to solve Equation (1) with a time step of 0.02 ms. In order to model the action potential during chronic AF remodeling, a combination of parameters sets from earlier publications (Courtemanche et al., 1999; Pandit et al., 2005; Zhang et al., 2005) was adopted. In particular, we down-regulated the maximal conductances of *I*_*to*_, and *I*_*CaL*_ by 85 and 74%, respectively, increased the maximal conductance of *I*_*K*1_ by 250%, increased the time constant for activation of *I*_*CaL*_ by 62%, shifted the fast *Na*^+^ inactivation curve by +1.6 mV, the *I*_*to*_ activation curve by +16 mV, and the *I*_*CaL*_ activation curve by −5.4 mV. Since we did not have experimental data of our own to model the condition of AF, we relied on disease representations that were available in literature to design the specific model for chronic AF remodeling. A detailed study of relevant scientific literature suggests that every diseased substrate is different and more often that not, there is a mixture of abnormal conditions, rather than clear partitioning between a disease condition “A” and a disease condition “B.” Thus, we find our choice of parameters for the AF remodeling well-justified. Furthermore, this choice is in line with Malki and Zlochiver (2018), who used a similar model for chronic AF remodeling in their studies.

**Figure 1:**
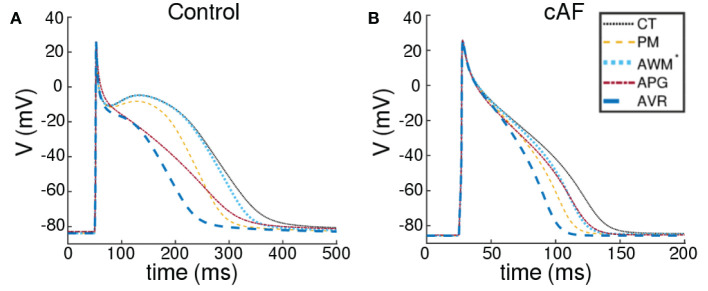
Action potentials obtained using region-specific models for different parts of the atria, under **(A)** control and **(B)** chronic AF (cAF) remodeled conditions. The dotted black line represents the AP from crista terminalis (CT), the dashed orange line represents the AP from the pectinate muscle region (PM); the dashed maroon line represents the AP from left and right atrial appendages (APG), and the thick dashed blue line represents the AP from the left and right atrioventicular regions (AVR). The rest of the atria, i.e., the Bachmann's bundle (BB), fossa ovalis (FO), pulmonary veins (PV), the isthmus of the right atrium (RIS) and the atrial working myocardium (AWM) is modeled using a single set of parameters. The corresponding AP is shown using a dotted blue line (AWM^*^).

**Figure 2:**
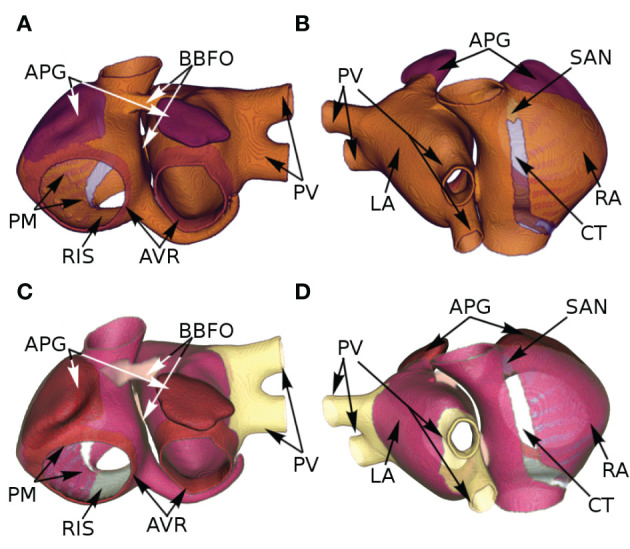
Segmentation of the atria according to AP-based **(A,B)** and conduction-based **(C,D)** heterogeneity, as assigned by Tobón et al. (2013).

We took into account the following regions of the atria (check **Figure 2** for reference): the crista terminalis (CT), Bachmann's bundle (BB), pectinate muscles in the right atrial free wall (PM), the fossa ovalis (FO), isthmus of the right atrium (RIS), the atrial appendages (APG), the atrio-ventricular regions (AVR), sinoatrial node (SAN), pulmonary veins (PV) and finally, the atrial working myocardium (AWM). To model the region-based heterogeneity of the atria, we adjusted the maximal conductances of specific ion channels. The adjustment was constrained by the condition that in switching from normal to remodeled atria, the APD_90_ values remained within ~ 10% deviation of the ones obtained by Tobón et al. (2013). The full list of maximal conductances is given in Table 1. Figure 1 shows a comparison between APs obtained from different regions of the atria, as produced using our model in both control and remodeled atria.

**Table 1:** Region-specific adjustments to the CRN model, with corresponding APD_90_ and CV at 37°*C*, in the control atria.

	**CT**	**BBFO**	**PM**	**PV**	**RIS**	**AWM**	**AVR**	**APG**
*G*_*K*1_ (nS/pF)	0.09	0.117	0.1188	0.117	0.117	0.117	0.117	0.09
*G*_*to*_ (nS/pF)	0.1652	0.1652	0.1652	0.1652	0.1652	0.1652	0.1652	0.0578
*G*_*Kr*_ (nS/pF)	0.0294	0.0294	0.0588	0.0294	0.0294	0.0294	0.1176	0.0324
*G*_*CaL*_ (nS/pF)	0.1238	0.1238	0.1238	0.1238	0.1238	0.1238	0.1176	0.0619
*APD*_90_ (ms)	307	284	242	284	284	284	183	253
D (cm^2^/ms)	0.009	0.0064	0.0064	0.0016	0.0013	0.0025	0.0025	0.0025
*CV* (cm/s)	142.5	117.75	117.75	54.75	48	69.75	69.75	71.25

Like Tobón et al. (2013), we considered differences in propagation velocities in different parts of the atria. Thus, the idea was to get regions with propagation speed as fast as 120−140 cm/s (PM and CT) coexist with regions of propagation, as slow as 25−45 cm/s (SAN and RIS). To this purpose, we extended our cell model to two dimensions (2D), whereby the regional CV could be factored in through the choice of the diffusion coefficient D. As in case of the region-specific values of the ion channel conductances, D, was determined based on the constraint that in switching from normal to remodeled atria, region-specific CV demonstrated acceptable resemblance to the ones reported by Tobón et al. (2013), in both normal and remodeled cases. In 2D, spatiotemporal evolution of *V* was modeled according to the following reaction-diffusion-type equation:

(3) $$\begin{array}{l} \frac{dV}{dt}=\nabla \cdot \left(D\nabla V\right)-\frac{I_{ion}+I_{stim}}{C_{m}} \end{array}$$

The temporal part was solved numerically using forward Euler method, as in single cells, whereas the Laplacian in the spatial part (D is a constant in 2D) was solved using centered finite difference technique, with a spatial resolution of 0.03 cm, which was fixed by the spatial resolution of the DTMRI data, used for the anatomically realistic simulations.

Next, we incorporated temperature scaling of parameters in this model, according to the Hodgkin and Katz formalism Hodgkin and Katz (1949), by either multiplying the rate constants α(37°*C*) and β(37°*C*) of the gating variables of specific ion channels (see below) by *Q*(*T*), or, by dividing the time constant τ(37°*C*) of these ion channels by *Q*(*T*) (in case the α and β values were unavailable).

(4) $$\begin{array}{l} \alpha \left(T\right)=\alpha \left(37{}^{\circ}C\right).Q\left(T\right) \end{array}$$

(5) $$\begin{array}{l} \beta \left(T\right)=\beta \left(37{}^{\circ}C\right).Q\left(T\right) \end{array}$$

(6) $$\begin{array}{l} Q\left(T\right)={\left[Q_{10}\right]}^{\frac{T-37{}^{\circ}C}{10{}^{\circ}C}} \end{array}$$

Th *Q*_10_ factors used for the different currents, were obtained from Malki and Zlochiver (2018) (see Table 2 below).

**Table 2:** Temperature scaling factors (*Q*_10_ values) for different ionic currents.

**Current**	***I*_*Na*_**	***I*_*to*_**	***I*_*Kur*_**	***I*_*Kr*_**	***I*_*Ks*_**	***I*_*CaL*_**
*Q*_10_	3.0	2.2	2.2	3.3	2.57	2.2

We studied RMP in single cells. To measure its temperature dependence, we started with the standard steady-state conditions of the CRN base model. We altered the maximal conductances of specific ion channels, as described in Table 1 and set ambient temperature to *T*_1_ ($5{}^{\circ}C<T_{1}<50{}^{\circ}C$). We then allowed the system to evolve in time for 10 s, in the absence of an applied stimulus. The minimum current (χ), required to elicit an AP at different temperatures, was measured in a manner, similar to RMP measurements, with the stimulus being applied at the end of 10 s, when the system was assumed to have reached a more-or-less steady state.

To measure restitution characteristics, we used a 2D strip domain containing 512×10 grid points. After allowing the system to stabilize for 5 s at a given ambient temperature *T*_1_, we initiated a train of 20 right-propagating plane waves (paced at 1.0 Hz), from the region *x* < 5 grid points, by applying current stimuli of strength 25 μA and duration 2.0 ms. After the application of the 10*th* stimulus, we let the system evolve freely for a brief period of time (the diastolic interval DI), and applied a final (11*th*) pulse of the same strength and duration. We repeated the procedure at $5{}^{\circ}C\leq T_{1}\leq 50{}^{\circ}C$ in steps of 5°*C*, for all considered regions of the atria. In each case, we measured APD_50_, APD_90_ and propagation speed (CV), from the wave of electrical activation, corresponding to the 11*th* stimulus.

Lastly, we studied the effect of change in temperature on scroll wave dynamics in the anatomically-realistic, electrophysiologically-detailed human atria. To construct the geometry and fiber structure, we used open-source DTMRI data available from Tobón et al. (2013). For a detailed description of how the DTMRI data was procured, segmented and processed, we refer the readers to Tobón et al. (2013). The irregular boundaries were handled by a phase field implementation (Fenton et al., 2005; Clayton and Panfilov, 2008), whereby, we embedded the atria in a rectangular parallelepiped grid structure composed of regular cubic nodes. We defined a field variable ϕ in this domain, such that *t* = 0, all points outside the atria are reset (ϕ(*x, y, z*) = 0) and all points belonging to the atria, are set (ϕ(*x, y, z*) = 1). Next, we evolved ϕ in space and time using a relaxation method (Equation 7) to smoothen the interface between the atrial boundary and the exterior.

(7) $$\begin{array}{l} \frac{\partial \phi }{\partial t}=\xi^{2}\nabla^{2}\phi -\frac{\partial G\left(\phi \right)}{\partial \phi } \end{array}$$

The parameter ξ determines the width of the interface, and *G*(ϕ) is a standard double-well potential:

(8) $$\begin{array}{l} G\left(\phi \right)=-\frac{{\left(2\phi -1\right)}^{2}}{4}+\frac{{\left(2\phi -1\right)}^{4}}{8} \end{array}$$

We used the spatial profile of ϕ, obtained after 500 iterations (with a spatial resolution of 0.03 cm and time step δ*t* = 0.02 ms), for studying electrical activity in the atria. At each node with ϕ ≠ 0, we integrated the temperature sensitive ionic models of the region-specific cardiomyocytes, that we developed within the first part of the paper. For the pacemaker, however, we used the AWM model with reduced CV, and a simple periodic step function to represent the nodal electrical activity from the designated region. Then, using an 18−point stencil, we solved (Equation 9), a modified version of Equation (3), to compute the spatiotemporal evolution of *V*.

(9) $$\begin{array}{l} \phi \frac{\partial V}{\partial t}=\nabla \cdot \left[D\phi \nabla V\right]-\phi \frac{I_{ion}+I_{stim}}{C_{m}} \end{array}$$

Note, that in these 3D anatomical models, the diffusion coefficient D is no longer a scalar constant, but a rank-3 tensor, whose components (*d*_*ij*_) are related to the fiber directions (*a*_*i*_), as follows:

(10) $$\begin{array}{l} d_{ij}=\left\{\begin{array}{cc} D_{⊥}+\left(D_{∥}-D_{⊥}\right)a_{i}a_{j} & i=j\\ \left(D_{∥}-D_{⊥}\right)a_{i}a_{j} & i\neq j \end{array}\right\} \end{array}$$

The atria were segmented according to AP-based heterogeneity as per Figures 2A,B. To incorporate conduction heterogeneity, the atria were segmented according to Figures 2C,D.

To compute the temperature field (*T*) in 2D and 3D simulations, we solved the time-dependent heat-transfer equation (Paul et al., 2014):

(11) $$\begin{array}{l} \left(\rho C\right)\frac{\partial T}{\partial t}=\nabla \cdot \left(k_{t}\nabla T\right) \end{array}$$

Here, ρ, *C*, and *k*_*t*_ represent, respectively, the density (1,050 kg/m^3^), specific heat capacity [4,219 J/(kgK)], and thermal conductivity [0.7 W/(mK)] of human cardiac tissue. For these simulations, we assumed that the temperature both outside the atria, and in the cavities, was 37°*C*.

## 3. Results

### 3.1. Single Cell Simulations

**Figure 3:**
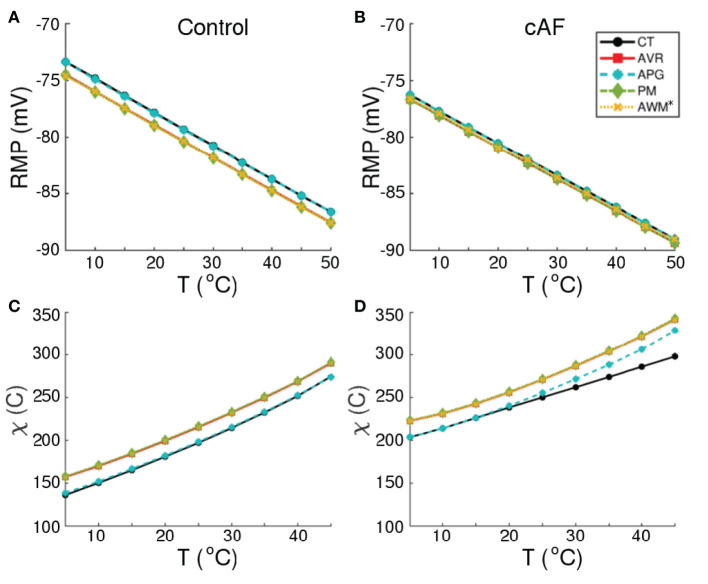
Temperature-dependence of RMP, and minimum stimulus (χ, in Coulombs) required to produce an action potential, in normal **(A,C)** and remodeled **(B,D)** cardiac cells, isolated from different regions of the human atria (CT, AVR, APG, PM, and AWM^*^).

Our simulations of electrical activity in cardiomyocytes isolated from different parts of the atria, show that RMP correlates negatively with ambient temperature for both normal (Control) and remodeled (cAF) conditions. This is shown in Figures 3A,B. As a direct consequence, at elevated temperatures (>48°*C*), cardiomyocytes from all regions of the atria become functionally inexcitable. Figures 3C,D illustrate the temperature dependence of the minimum amount of electric charge (χ, measured in Coulombs) required by the cell to produce an action potential in both Control and cAF cases. At *T* ≳ 48°*C*, irrespective of the value of χ, one cannot obtain action potentials because the RMP is extremely low, leading to complete inactivation of the fast *Na*^+^ channels, which are responsible for the primary excitability of the cell. The applied excitation then dissipates exponentially, as in a decaying RC-circuit. Exposure to such high temperatures for up to several seconds can even lead to permanent damage of the cell membrane, with complete loss of excitability (Kolandaivelu et al., 2010).

**Figure 4:**
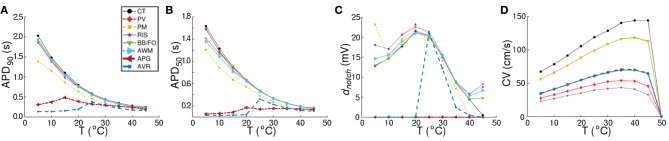
Comparison of region-specific temperature-dependencies of AP characteristics **(A)** APD_90_, **(B)** APD_50_, and **(C)** notch depth (*d*_*notch*_), and **(D)** conduction velocity (CV) under normal (Control) conditions.

Our model illustrates (in consonance with what is known about human atrial cardiomyocytes), that at body temperature (37°*C*), the action potential is longest (~ 307 ms) in the CT region, with a pronounced notch around ~ −10 mV. The AP is shortest in the AVR region (~ 183 ms), with a triangular morphology and complete disappearance of the notch. AP morphology is also triangular in the AWM region, whereas, in APG and PM, a short plateau is visible. AP morphology exhibits dramatic dependence on temperature. Comparative AP traces from different parts of the atria at different temperatures (5−50°*C*) reveal that, the qualitative effect on AP morphology is most pronounced in the CT region, where lowering of temperature is accompanied by a prolongation of the plateau phase. The least dependence on temperature is observed in the APG and AVR regions, where the AP is generally less affected, except within the temperature range 20°*C* ≲ *T* ≲ 30°*C*, where alternans appears. For quantitative comparison, temperature-dependency curves for APD_90_, APD_50_ and notch depth (*d*_*notch*_) are presented in Figures 4A–D. Clearly, the effect of temperature on APD_90_ is maximum in the CT region (see Figure 4A), followed by BBFO, AWM, PV and RIS (comparable with PV). In APG and AVR, while a decrease in *T* from 50°*C* shows an initial increase in APD_90_ similar to other atrial regions, below a critical temperature (~ 25°*C* for AVR and ~ 15°*C* for APG), APD_90_ decreases with decrease in *T*.

We observed that at these critical temperatures, the single-cell models for APG and AVR showed alternans. Similar trends were observed with APD_50_ (see Figure 4B), although, in this case the APG model remained approximately independent of temperature at *T* ≳ 20°*C*. Below ~ 20°*C*, APD_50_ decreased with decrease in *T*. Finally, in Figure 4C, we illustrate the temperature-dependence of *d*_*notch*_. Our results demonstrate that for all regions except APG and AVR, the AP exhibits a notch at body temperature (37°*C*). The notch depth grows as *T* is decreased down to ~ 20°*C*. Below this temperature, *d*_*notch*_ decreases monotonically except at PM, PV, and RIS, where, it goes through a minimum at ~ 10°*C*. When heated to temperatures above 37°*C*, *d*_*notch*_ decreases everywhere except at APG and AVR. Here, heating can be associated with a gradual triangulation of the AP morphology. In all regions except CT, *d*_*notch*_ goes through a second minimum at ≃40°*C*, followed by a slow increase with further rise in temperature. The presence of a deep notch indicates the termination of *I*_*Na*_, followed by a transient depolarization in the presence of reduced *I*_*CaL*_. Our data shows that the *d*_*notch*_ increases with mild hypothermia, but decreases as the temperature is reduced further. Lowering of temperature to below 20°C results in a decrease in the rate of depolarization and AP amplitude, together with a decrease in the transient repolarization by *I*_*to*_ and an increase in APD. The AP from APG is always triangular, i.e, *d*_*notch*_ = 0 mV at all temperatures, whereas the notch disappears from AVR APs at *T* ≲ 20°*C*. At this temperature, *d*_*notch,AVR*_ ≈ *d*_*notch,RIS*_.

### 3.2. Conduction Velocity Studies

A study of temperature-dependence of conduction velocity (CV), as measured from different parts of the atria, showed that CV increased linearly with increase in *T* for 5°*C* ≲ *T* ≲ 25°*C*. Above 25°*C*, CV still increased with T, but at a rate that became progressively slower. Finally, above 40°*C*, CV decreased rapidly to 0 by 50°*C*, at which point, action potentials were no longer formed. This is shown in Figure 4D. Normalization of CV by its value at 37°*C* yielded a family of curves, each with slope ~ 1.02. This is interesting, given that the models used to simulate the different regions of the atria, make use of different electrophysiological parameters. The temperature-dependence of APD_90_ and CV in single cardiac cells and strip domains constructed from cells belonging to different regions of the atria are tabulated in Table 3.

**Table 3:** Comparison between region-specific APD_50_, APD_90_, and CV values for control (C) and remodeled (R) atria, at 37°*C*, from tissue simulations.

	**Condition**	**CT**	**BBFO**	**PM**	**PV**	**RIS**	**AWM**	**AVR**	**APG**
APD_50_ (ms)	C	223.74	218.58	184.02	220.02	218.82	221	134.24	154.64
APD_50_ (ms)	R	73.3	66	60.4	62.8	61.4	65.3	52.76	60.3
APD_90_ (ms)	C	319.48	294.4	253.62	296.52	296.38	296.44	213.98	276.1
APD_90_ (ms)	R	107.4	92.5	85.7	90.6	90	91.7	77.1	94.2
CV (cm/s)	C	142.5	117.75	117.75	54.75	48	69.75	69.75	71.25
CV (cm/s)	R	137.25	113.6	113.25	51.75	40.9	67.5	67.5	68.6

### 3.3. APD and CV Restitution

**Figure 5:**
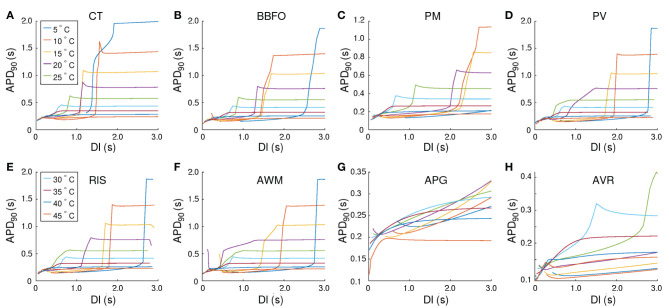
Temperature-dependence of APD_90_ restitution curves recorded from (15.36 × 3 *mm*) tissue strip containing cells from **(A)** CT, **(B)** BBFO, **(C)** PM, **(D)** PV, **(E)** RIS, **(F)** AWM, **(G)** APG, and **(H)** AVR, respectively. Restitution was measured using the dynamic S1-S2 protocol, whereby, each tissue strip was first allowed to reach a steady state at a given temperature (5° < *T* < 45°*C*). Then a series of 20 S1 pulses were applied at the left end of the strip, at a pacing frequency of 1 *Hz*. Finally, a 21*st* stimulus (S2) was applied in a similar manner, but after a variable diastolic interval (DI). Our results indicate the occurrence of a phenomenon similar to a first order state transition, at very low temperatures in most atrial regions. At high (*T* > 40°*C*), the APD restitution curves exhibit a negative slope.

**Figure 6:**
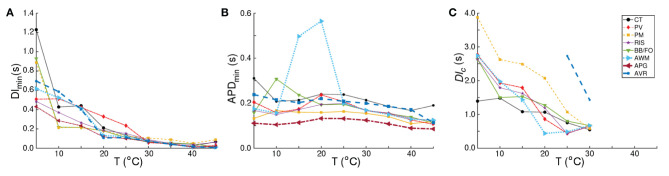
Analysis of the APD restitution curves for all atrial regions. Temperature-dependence of **(A)** DI_*min*_, **(B)** APD_*min*_, and **(C)** *DI*_*c*_, as measured from CT, BBFO, PM, PV, RIS, AWM, APG, and AVR. DI_*min*_ shows a general hyperbolic decrease with increase in temperature. APD_*min*_ on the other hand, increases slightly with decrease in temperature, reaching a maximum around 20°C and then decreasing again to approximately its value at 37°C. The sudden large deviation in APD_*min*_ around 15−20°*C* for AWM arises from the atypical behavior of its corresponding APD restitution curve (see Figure 5F). DI_*c*_ increases almost linearly with decrease in temperature for each region, although the rate of increase is region-dependent.

In our studies with single atrial cells, we noted that electrical properties of the cell at any given temperature, depended heavily on the frequency with which the cell was stimulated. In particular, for regions such as CT, BBFO, AWM, PV and RIS, which show APs ~ O(2 s) at 5°*C*, it is important to understand how the sub-cellular processes cope with slow and fast periodic electrical stimulation. Therefore, we investigated the temperature-dependence of restitution. Our results are presented in Figures 5A–H. We observed that, although the APD restitution curve for CT at 37°*C* follows the standard trend, with maximum slope <1.0, the restitution behavior is affected remarkably at high and low temperatures. In particular, at *T* < 37°*C*, the APD restitution curve shows an initial slow rising phase (with very small positive slope). Then, at a critical diastolic interval (*DI*_*c*_), the slope changes dramatically, as if the restitution behavior is undergoing some sort of state transition. Finally, at large DI, APD saturates to a large value. Figures 6A–C show detailed analysis of the APD restitution curve for CT at different temperatures.

The APD resitution curve for AWM shows similar trends as CT, except that at temperatures ~ 15−20°*C* the CT-like curves are preceded by an additional rapid phase, in which the restitution curve has a large negative slope. The detailed analysis of the APD restitution curve for AWM is presented in Figures 6A–C. APD restitution trends similar to CT were also observed in PM, PV, BBFO and RIS regions, although, as presented in the analysis plots of Figure 6, the maximum value of the APD reached by a system, and the steepness of the slope during the possible state transition, increased with decrease in temperature.

Finally, the APD restitution curves of APG and AVR exhibited behaviors that were different from the rest of the atria. In APG, at *T* < 35°*C*, APD saturation did not occur, even at DI = 3,000 ms. The curves then showed an initial phase with a small negative slope, which gradually increased to a positive value. Hereafter, APD kept increasing with DI. At *T* ≳ 35°*C*, the APD restitution curves followed the standard trend with saturation of APD at large DI. As for AVR, the APD restitution curves exhibited trends similar to APG at all temperatures, except ~ 25−30°*C*. In this range of temperatures, the APD restitution curves showed signatures of a possible state transition.

**Figure 7:**
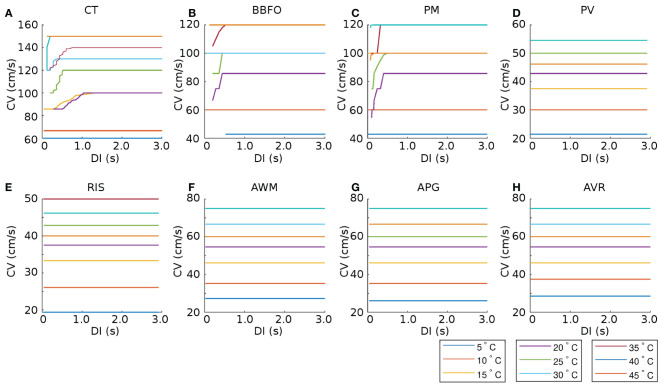
Temperature-dependence of CV restitution curves recorded from (15.36 × 3 *mm*) tissue strips containing cells from **(A)** CT, **(B)** BBFO, **(C)** PM, **(D)** PV, **(E)** RIS, **(F)** AWM, **(G)** APG, and **(H)** AVR, respectively. Restitution was measured using the dynamic S1-S2 protocol (see section 2 for details). Our results show that CV is more or less independent of DI for most regions (PV, RIS, AWM, APG, and AVR), at all temperatures. However, in some regions (CT, BBFO, and PM), CV exhibits initial fluctuations at low DI at moderate temperatures.

In most atrial regions, the CV restitution curves exhibited a temperature-dependency. However, CV, itself, appeared to be independent of DI in all regions except CT, PM, and BBFO. These data are presented in the plots of Figure 7.

### 3.4. Effect of Cooling the Anatomically Realistic Human Atria

**Figure 8:**
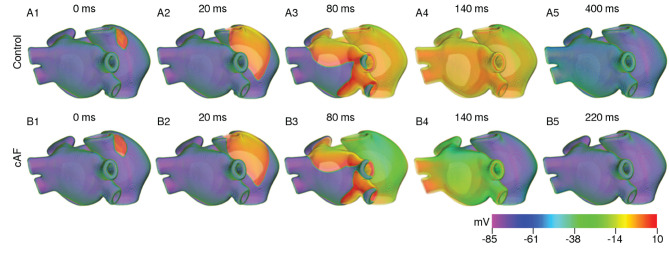
Propagation of a plane wave through the human atria at normal body temperature (37°C).

Based on our findings at the single cell level and with strips, we investigated the effect of cooling on anatomically realistic, electrophysiologically detailed human atria, in the presence of scroll waves. First, we simulated wave propagation in the 3D atrial model at normal body temperature (37°*C*). We looked at the propagation patterns of electrical signals originating at the SAN, and compared our findings with those of Tobón et al. (2013). As illustrated in Figure 8, the electrical signal initiated at the SAN propagated down the right atrium with a triangular wavefront, due to large differences in conduction velocities between the CT and AWM regions. In our model of the normal heart, the time to full depolarization of both atria was ~ 140 ms, which is in line with the observations of Tobón et al. (2013). In the case of cAF, due to the reduction in signal wavelength, repolarization of the right atrium sets in before the left atrium is fully depolarized. These results are presented in Figure 8. A demonstration of plane wave propagation in anatomical atria under normal and cAF-remodeled conditions is presented in Supplementary Video 1.

**Figure 9:**
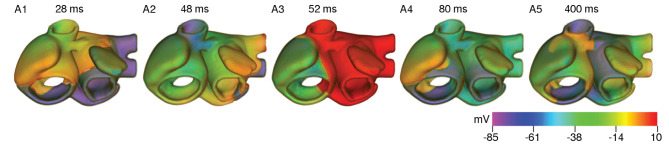
Formation of a pair of counter-rotating scroll waves by S1-S2 cross-field protocol at 37°*C* under cAF-remodeled conditions.

To study the effect of temperature variation on arrhythmias, we produced a pair of counter-rotating scroll waves in the remodeled human atria (see Figure 9) by applying S1-S2 cross-field protocol. We did not initiate the scroll in the control atria because we assumed that arrhythmias are unlikely to occur in healthy atria. Technically, however, scroll wave initiation should be possible in the control atria, following the same protocol. The S1 wave originated at the SAN. When its waveback had propagated all the way up to the AVR, and half the mass of the excited tissue in the atria had recovered from refractoriness, we applied the S2 stimulus, perpendicular to the S1 wave. This resulted in the production of a pair of counter-rotating scroll waves, of which, one (centered in the right APG region) became the principal driver of the rotational activity, whereas the other anchored to one of the caval veins and converted itself into anatomical reentry. A demonstration of the formation of a scroll wave from the point of application of the S2 stimulus is shown in Supplementary Video 2.

**Figure 10:**
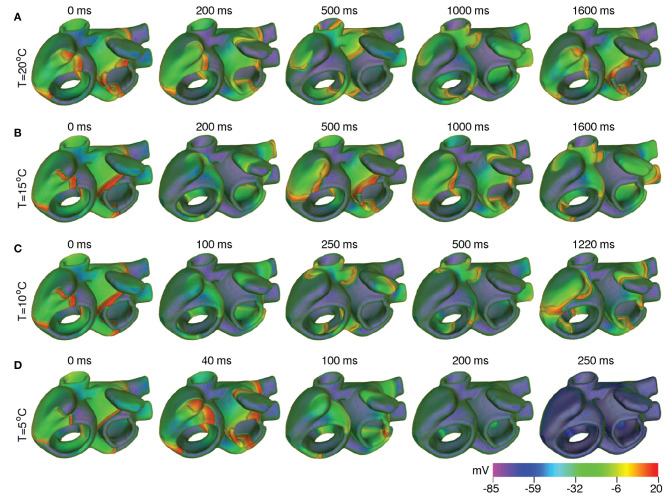
Scroll wave dynamics in the remodeled human atria, under the effect of global cooling at temperatures **(A)** 20°*C*, **(B)** 15°*C*, **(C)** 10°*C*, and **(D)** 5°*C*. Moderate cooling (15−20°*C*) leads to stabilization of the initial scroll wave, with reduced period. At ~ 10°*C*, the scroll wave breaks up. The atria then supports multiple scroll waves that maintain a state of sustained AF. Severe cooling (~ 5°*C*) leads to suppression of the scroll wave, within 200 ms.

In order to test the effect of cooling on the spatiotemporal dynamics of scroll waves in the atria, as a first approach, we applied uniform cooling to the entire thickness of the atrial wall, in all parts of the atria. As proven by our simulations with the strip domain, different atrial regions responded differently to the same decrease in ambient temperature. A direct consequence of this heterogeneous electrophysiological response, is the increased heterogeneity of AP characteristics, which happens with reduction in temperature. At moderately low temperatures (20°*C* < *T* < 35°*C*), the arrhythmia stabilized via establishment of a stable core of the rotating scroll wave (see Figure 10A). At lower temperatures (10°*C* < *T* ≲ 20°*C*), alternans in some parts of the atria promoted degeneration of a single scroll wave into multiple self-sustained wavelets that coordinate to maintain a state of sustained AF (see Figures 10B,C). Interestingly, further decrease in temperature to ~ 5°*C* led to suppression of the scroll waves, due to substantially reduced excitability of the tissue, which could no longer sustain the scroll wave (see Figure 10D). In this situation, the drop in temperature caused the filament of the primary scroll wave to meander away from the right APG region into the right AVR, where it got absorbed at the boundary. This further led to termination of the reentrant wave around the caval vein, which received its driving force from the scroll wave in the right APG. Thus, severe cooling for very short duration, within 200 ms as shown in our study, can form the basis for a new approach to arrhythmia management and therapy. These results are shown in Supplementary Video 3. As demonstrated by our single cell and tissue level studies (effects of temperature on APD and wavelength, respectively) the effect of cooling is more pronounced in the control atria. Thus, if a scroll wave was to be initiated there, lowering the temperature would result in faster termination of the arrhythmia, at temperatures that are higher than in the remodeled case.

## 4. Discussion

We performed an extensive, systematic thermal characterization of the anatomically-realistic, electrophysiologically-detailed human atria. Our study gives novel insights into the crucial role of temperature in the determination of spatiotemporal dynamics of scroll waves in the atria, when subjected to cooling at different temperatures. By constructing strip domains that contain cardiomyocytes specific to different atrial regions (e.g., CT, PM, PV, BBFO, RIS, AWM, APG, and AVR), we demonstrated how lowering of temperature from 37°*C* (normal body temperature) to ≃20°*C* (room temperature) could already induce alternans in different regions of the atria, thereby facilitating the development of a proarrhythmic substrate. Our results indicate that further cooling (≃10°*C*) could lead to break up of stable rotating scroll waves into multiple wavelets that were capable of sustaining themselves through mutual interaction. Finally, severe cooling (~ 5°*C*) for very short duration (~ 200 ms) could successfully terminate all electrical activity in the atria, through destabilization caused by sudden, substantial decrease in tissue excitability.

Very little is known about the effects of temperature on spiral and scroll wave dynamics in cardiac tissue, and even less so, in human subjects. In our study at the single cell and tissue level, we observed depolarization of the RMP with decrease in temperature. Similar increase was observed by Fedorov et al. (2005) in hearts of rabbits and ground squirrels, who also reported a 10-fold decrease in CV, and increased excitation threshold in going from 37 to 3°*C*. In contrast, our studies revealed that in human atria, the decrease in CV (ΔCV) could reach up to a maximum of ~ 3 − *fold* in the CT region for a decrease in temperature from 37 to 5°*C*. ΔCV was found to be less in other regions of the atria, for the same decrease in temperature. We also observed an increased excitation threshold, with decrease in temperature, consistent with the findings of Fedorov et al. (2005).

In another experimental study using hearts of rabbits and ground squirrels, Egorov et al. (2012) reported observing spatially discordant alternans at temperatures as low as 17°*C*. Similar alternans was also observed in canine ventricular preparations by Fenton et al. (2013), who provide a quantitative description of emergent proarrhythmic properties of restitution, conduction velocity, and alternans regimes as a function of temperature. We observed APD alternans at low temperatures (15−20°*C*) in specific regions of the atria, which led to the promotion of wavebreaks in the simulations involving anatomically realistic atria. Furthermore, we observed similar temperature-dependency trends of the APD restitution curves as Fenton et al. (2013) at lower temperatures, e.g., the sharp first order phase transition-type change in the APD value about a critical DI, which Fenton et al. (2013) identify as a sigmoidal shape of the APD restitution curve, and associate with a regime where alternans in suppressed. Here, we characterize human atrial electrophysiology, by comparing what parts when temperature is reduced globally. In this context we would like to emphasize that even if some parts of the atria, such as CT, BBFO and PM show nonlinear restitution at low temperatures, neighboring regions such as AWM, APG, and AVR remain unaffected. In principle, it would be nice to establish markers of spiral wave susceptibility/interruption based on the characterization of APD and CV restitution. However, here we refrain from doing so because of insufficient evidence to justify such quantification. Moreover, our simulations of restitution were performed in 2D, which is vastly different from the anatomical geometry: the latter has its own contribution to promoting or suppressing the break-up of scroll waves. Thus, we believe our results to form an important first step in the temperature-based characterization of the human atrial electrophysiology, but further validation is required before we can speculate on the significance or lack thereof, of these results in the real environment.

Dedicated studies by Filippi et al. (2014), into the mechanisms that induce fibrillation during hypothermia. reveal that prolongation of APD is accompanied by comparable reduction in CV at low temperatures, resulting in effective reduction in spatial wavelength and excitability. Thus, fast pacing frequencies, as those observed with spiral waves in the system, can induce wavebreaks or sustained reentry at low temperatures. Our studies are consistent with these findings. We observed that, a certain range of temperatures (10−20°*C*) provided the optimal substrate for onset of wavebreak in the remodeled human atria. At these temperatures, the wavelength decreased just enough to simultaneously fit multiple scroll waves in the whole atria. The breakup and regeneration of scroll waves occurred mostly around the PV regions. This is in line with the findings of Chen et al. (2003), who reported the occurrence of triggered electrical activity in single pulmonary vein cardiomyocytes isolated from rabbit hearts, and identified them as possibile underlying causes of proarrhythmicity in the atria. However, contrary to Chen et al. (2003), who observed such behavior only at high temperatures, our observations were at moderate to low temperatures, which made global cooling ineffective even at 10°*C*. Cooling below 5°*C*, however, delivered sufficient destabilization to the scroll wave, thereby pushing it toward an inexcitable boundary of the domain, favoring termination.

To study the effect of temperature in the whole atria, we performed simulation studies with global cooling. Previous studies by Malki and Zlochiver (2018) in simulated 2D human atria explored the possibility to employ spatial gradients of temperature to enforce spiral wave drifting. Such gradients, though effective in directing the movement of a spiral wave through 2D media, are actually extremely difficult to impose in a real non-uniformly curved 3D system, such as the anatomical atria. In addition, there is the issue of intrinsic electrophysiological heterogeneity that Malki and Zlochiver (2018) neglect in their proof-of-principle study, but which has huge influence on electrical wave propagation, as was demonstrated earlier by Tobón et al. (2013) and now, in our present study. In this context, it must be also highlighted that our study uses only one orientation dataset. Thus, the results that we obtain are valid for this particular anatomy of the atria. We also induced the scroll wave by applying only one type of induction protocol. Although, from nonlinear dynamics we know that the spatiotemporal evolution of scroll waves depends only on the properties, topology and excitability of the substrate, and not so much on the induction protocol itself, using different induction protocol would change the location of the scroll core. In this study, we were interested to see how temperature changes affect the ability of the atrial muscle to sustain scroll waves. Thus, we chose a configuration of the scroll which allowed it to rotate stably for 10 s at 37°C. We believe that further simulations using different atrial anatomies and induction protocols are not required to prove this basic point that we have tried to establish in our study.

In another landmark study, Yamazaki et al. (2012) demonstrated for the first time, the possibility to control ventricular arrhythmias in rabbit hearts by applying local cooling. Such studies form the next step in the development of thermal-based arrhythmia management strategies. In particular, it would be interesting to identify specific target regions within the atria, which, when cooled, could lead to highest success rate in removing arrhythmias by destabilization through sudden decrease in tissue excitability. Independently, Guill et al. (2012) studied the effects of local epicardial cooling/heating on the complexity of ventricular arrhythmias. They found that cooling induced deceleration of VF which led to increase in the complexity of VF activation patterns, whereas, heating induced acceleration of VF. In our 3D simulations with the anatomically realistic atria, we have not explored the effects of local heating/cooling. As a first approach, we applied low temperature uniformly to all parts of the atrial wall. In the real heart, blood perfusion and equilibriation with the surrounding body temperature prevents this from happening at least immediately after the cooling is applied. Thus, ideally, one should consider the application of the low temperature on the outer or inner surface of the atrial wall and let the system build its own temperature profile. Given that, cooling down to temperatures like 5 and 10°*C* have such contrasting effects on scroll wave dynamics, one can argue that surface cooling at 5°*C* may not be sufficient to terminate the arrhythmia. However, the atrial wall is very thin. Therefore, elimination of activity from several shells of the atrial wall may actually be sufficient to cause removal of the scroll waves from the entire atria. Furthermore, considering the temperature-dependency of the atrial geometry itself, Fedorov et al. (2008) reported a possible variation, with temperature, of the anisotropy ratio of fibers in the hearts of rabbits and ground squirrels. In our studies, we did not factor in the component of a temperature-dependent anisotropy ratio, owing to the lack of availability of experimental data to base our model on. These paths remain to be explored in future studies. Useful insights can be gained by repeating these studies with scroll waves initiated at different sites in the atria. This can be done by applying the S2 wave at different times after the passage of the S1 wave or by using induction protocols other than S1-S2. Another option would be to introduce natural cellular heterogeneity within the atria (i.e., by varying the maximum channel conductance of each cell in the domain within acceptable limits) or by introducing diffuse fibrotic tissue into the atria. Performing statistics based on several such simulations would give us robust results for assessing the future potential of the cooling method in arrhythmia treatment and therapy. However, such simulations are extremely computationally expensive and beyond the scope of this manuscript.

Having said that, it should be mentioned that in real human atrial tissue samples, AP generation or atrial contraction are not seen to occur at the tissue level at temperatures below 20°C. Our results are slightly different. A possible explanation could be that the mathematical models that we use to study the electrophysiological properties at organ level, are not sufficiently accurate, quantitatively, such low temperatures. Thus, the next step should be to perform detailed measurements of current-voltage characteristics and restitution curves at the cellular level, and fit the numerical model to these experimental data. Nevertheless, our studies confirm that cooling the heart to low temperatures for very short periods of time should be sufficient to trigger this effect. Once the atrial tissue loses its ability to respond with AP, no arrhythmia can sustain. Thus, cooling the heart may have potential applications in engineering medical devices that can substitute high voltage (high energy) shocks produced by conventional defibrillators, with low temperature (low energy) alternatives.

## Data Availability Statement

The original contributions presented in the study are included in the article/Supplementary Material, further inquiries can be directed to the corresponding author/s.

## Author Contributions

RM performed 3D simulations and drafted the manuscript. AM performed single cell and 1D simulations. YW visualized data and aided 3D simulations. RM, AP, EB, and YW developed the concept, performed data analysis, and reviewed the manuscript. All authors contributed to the article and approved the submitted version.

## Conflict of Interest

The authors declare that the research was conducted in the absence of any commercial or financial relationships that could be construed as a potential conflict of interest.

## Publisher's Note

All claims expressed in this article are solely those of the authors and do not necessarily represent those of their affiliated organizations, or those of the publisher, the editors and the reviewers. Any product that may be evaluated in this article, or claim that may be made by its manufacturer, is not guaranteed or endorsed by the publisher.
