# Supplementary material for: Electrophysiological Characterization of Human Atria: The Understated Role of Temperature
Source: Front Physiol. 2021 Jul 23;12:639149. doi: 10.3389/fphys.2021.639149 (PMC8346027; doi:10.3389/fphys.2021.639149)
Supplement: Supplementary file 4 [file Data_Sheet_1.pdf]

Supplementary Material

1 SUPPLEMENTARY VIDEOS

Video 1: The propagation of plane waves through the atria under normal and cAF-remodeled conditions. The wave initiates at the sinoatrial node (seen clearly in View 1) and propagates anisotropically down the atrial wall. Propagation is fastest down the CT region. Thus the wavefront takes a triangular shape. In the Control case, excitation of the atria is uniform and global. However, in the cAF-remodeled case both atria are never fully excited at the same time. The videos show volume plots of the transmembrane potential with 50% regulated transparency (to filter out the background). Such filtering, unfortunately resulted in a visual artefact on the left atrial appendage. It apparently looks like it is excited at all times. However, this is not the case.

Video 2: Formation of a scroll wave using S1-S2 cross-field protocol. The video starts at the instant when the S2 stimulus is applied at the back of the S1 wave. The applied stimulus results in the initiation of two counter-rotating scroll waves.

Video 3: Scroll wave dynamics in the presence of cooling. Our studies show that wave termination is possible if the atria are cooled at $5°$C. At higher temperatures, cooling causes drastic reduction in wavelength of the spiral (from front to back), together with reduction in conduction velocity and rotation frequency. However, termination does not occur.
